# Circulating tumor DNA as a biomarker for monitoring early treatment responses of patients with advanced lung adenocarcinoma receiving immune checkpoint inhibitors

**DOI:** 10.1002/1878-0261.13090

**Published:** 2021-09-25

**Authors:** Paul van der Leest, Birgitta Hiddinga, Anneke Miedema, Maria L. Aguirre Azpurua, Naomi Rifaela, Arja ter Elst, Wim Timens, Harry J. M. Groen, Léon C. van Kempen, T. Jeroen N. Hiltermann, Ed Schuuring

**Affiliations:** ^1^ Department of Pathology University of Groningen University Medical Center Groningen The Netherlands; ^2^ Department of Pulmonary Diseases University of Groningen University Medical Center Groningen The Netherlands

**Keywords:** ctDNA, droplet digital PCR, ICI treatment response monitoring, NSCLC, PD‐L1

## Abstract

Immunotherapy for metastasized non‐small‐cell lung cancer (NSCLC) can show long‐lasting clinical responses. Selection of patients based on programmed death‐ligand 1 (PD‐L1) expression shows limited predictive value for durable clinical benefit (DCB). We investigated whether early treatment effects as measured by a change in circulating tumor DNA (ctDNA) level is a proxy of early tumor response to immunotherapy according to response evaluation criteria in solid tumors v1.1 criteria, progression‐free survival (PFS), DCB, and overall survival (OS). To this aim, blood tubes were collected from advanced‐stage lung adenocarcinoma patients (*n* = 100) receiving immune checkpoint inhibitors (ICI) at baseline (t_0_) and prior to first treatment evaluation (4–6 weeks; t_1_). Nontargetable (driver) mutations detected in the pretreatment tumor biopsy were used to quantify tumor‐specific ctDNA levels using droplet digital PCR. We found that changes in ctDNA levels were strongly associated with tumor response. A > 30% decrease in ctDNA at t_1_ correlated with a longer PFS and OS. In total, 80% of patients with a DCB of ≥ 26 weeks displayed a > 30% decrease in ctDNA levels. For patients with a PD‐L1 tumor proportion score of ≥ 1%, decreasing ctDNA levels were associated with a higher frequency a DCB (80%) and a prolonged median PFS (85 weeks) and OS (101 weeks) compared with patients with no decrease in ctDNA (34%; 11 and 39 weeks, respectively). This study shows that monitoring of ctDNA dynamics is an easy‐to‐use and promising tool for assessing PFS, DCB, and OS for ICI‐treated NSCLC patients.

## Graphical abstract

Predictive markers to reliably monitor response in patients with lung adenocarcinoma treated with immune checkpoint inhibitors are currently lacking. Here, we show that tumor‐informed monitoring of a single bloodborne mutation using droplet digital PCR enabled the identification of early disease progression and durable treatment responses. The association between patient survival and changes in mutant circulating tumor DNA (ctDNA) levels was unrelated to programmed death‐ligand 1 expression. Altogether, our data show that ctDNA dynamics could offer a promising cost‐effective approach to monitor patients with metastasized lung adenocarcinoma.

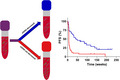

**Abbreviations**
ccfDNA: circulating cell‐free DNA
CD8+: cluster of differentiation 8 positive
CI: confidence interval
CR: complete response
CT: computed tomography
ctDNA: circulating tumor DNA
DCB: durable clinical benefit
ddPCR: droplet digital PCR
ECOG PS: Eastern Cooperative Oncology group performance‐status score
*EGFR*
: epidermal growth factor receptor
FFPE: formalin‐fixed paraffin-embedded
HR: hazard ratio
ICI: immune checkpoint inhibitors
*KRAS*
: kirsten rat sarcoma viral oncogene homolog
*MET*
: mesenchymal epithelial transition factor
NSCLC: non-small-cell lung cancer
OS: overall survival
PD: progressive disease
PD-L1: programmed death-ligand 1
PET/CT: positron emission tomography/computed tomography
PFS: progression-free survival
*PIK3CA*
: phosphatidylinositol-4,5-bisphosphate 3-kinase catalytic subunit alpha
PR: partial response
RECIST v1.1: response evaluation criteria in solid tumors version 1.1
SD: stable disease
*STK11*
: serine/threonine kinase 11
*TP53*
: tumor protein p53
TPS: tumor proportion score

## 1 Introduction

Treatment with immune checkpoint inhibitors (ICI) for advanced non‐small‐cell lung cancer (NSCLC) patients without targetable genetic alterations demonstrated long‐lasting therapy response and OS in selected patients [1, 2, 3, 4, 5]. Programmed death‐ligand 1 (PD‐L1) protein expression in the pretreatment tumor tissue determines eligibility for immunotherapy targeting PD‐1 or PD‐L1 inhibitors with or without chemotherapy. First‐line treatment with pembrolizumab is currently standard of care for patients with advanced NSCLC. However, even in patients with tumors having a high PD‐L1 expression (≥ 50% of tumor cells), a DCB of treatment is achieved in less than half of the cases [3, 6, 7]. Nivolumab monotherapy as treatment beyond first line resulted in 4‐year OS of 14% (95% confidence interval [CI]: 11–17%) for all patients (*n* = 664), 19% (95% CI: 15–24%) for those with at least 1% PD‐L1 expression, and 11% (95% CI: 7–16%) for those with less than 1% PD‐L1 expression [4]. Although eligibility criteria for immunotherapy are defined, there is an urgent demand for improved predictive and prognostic biomarkers that define which patients benefit from treatment. The ability to identify nonresponders at an early stage of ICI treatment could avoid severe toxicities associated unnecessary continuation of ICI treatment and reduce the financial burden on the healthcare system.

Solely relying on tumor PD‐L1 expression has proven clear limitations to accurately predict tumor response assessment by response evaluation criteria in solid tumors (RECIST) v1.1 criteria [8]. Furthermore, early on‐treatment radiologic assessment of tumor response cannot always predict durability of response because patients with initial pseudoprogression or stable disease (SD) may have durable responses comparable to patients who do have a radiological tumor response [4]. Therefore, a biomarker that better predicts or can monitor treatment effects for individual patients, alone or in combination with PD‐L1, is increasingly demanded [9]. Recent studies showed that monitoring the circulating tumor‐derived DNA (ctDNA) fraction in the circulating cell‐free DNA (ccfDNA) in plasma samples, as a surrogate for biological tumor response, correlates with individual early tumor responses and clinical outcome to treatment in several cancer types [10, 11], including NSCLC patients treated with ICI using expensive and complex next‐generation sequencing (NGS) methodologies on serial plasma ccfDNA [12, 13, 14, 15, 16].

Droplet digital PCR (ddPCR) analysis of plasma ctDNA is routinely used for clinical applications to detect targetable mutations in epidermal growth factor receptor (*EGFR*) [17, 18, 19], *KIT* [20], and *BRAF* [21, 22], with an analytical sensitivity of 0.1–0.01% and specificity > 99% [19, 20, 22, 23]. Here, we focused on a sensitive ddPCR test to monitor changes in ctDNA in plasma from advanced lung adenocarcinoma patients receiving single‐agent ICI. For this study, the target ctDNA was selected from the Pathology archives that reported on clinically relevant mutations determined by NGS analysis of the primary tumor in routine clinical practice. Patients with tumors harboring a nontargetable somatic mutation such as pathogenic mutations in kirsten rat sarcoma viral oncogene homolog (*KRAS*), and who were therefore treated with single‐agent ICI, were prospectively included. In addition to patients with *KRAS* mutations, patients with non‐*KRAS*‐mutated tumors (e.g., *BRAF* and phosphatidylinositol‐4,5‐bisphosphate 3‐kinase catalytic subunit alpha [*PIK3CA*] mutations) were included to rule out *KRAS* mutation‐specific observations. To date, only three other studies with relatively small cohorts of advanced NSCLC patients treated with ICI selected tumor‐informed nontargetable somatic mutations for monitoring ctDNA levels using a single‐gene assay [24, 25, 26]. Here, we investigated changes in ctDNA levels as a proxy of early tumor response to ICI for progression‐free survival (PFS), DCB, and OS in cohort of 100 patients with advanced lung adenocarcinoma using this approach.

## 2 Materials and methods

### 2.1 Patient selection

Patients were recruited between October 2015 and November 2019. In total, 100 patients with advanced adenocarcinoma receiving ICI treatment were eligible for this study. Mutation analysis via NGS of the pretreatment formalin‐fixed paraffin‐embedded (FFPE) tissue biopsies was performed in the routine diagnostic setting. These results were available for this study. Follow‐up data for all patients were obtained up to the database lock (October 9, 2020). Eligibility criteria were ≥ 18 years of age, Eastern Cooperative Oncology Group performance‐status score (ECOG PS) ≤ 1, advanced‐stage adenocarcinoma and measurable disease assessed by means of computed tomography (CT) according to RECIST v1.1 [27]. This study is a larger cohort based on CA209‐759 study (NTR 6158) and was approved by the Medical Ethical Committee (METc, 2010/109) of the University Medical Center Groningen (UMCG). The study methodologies were conformed to the standards set by the Declaration of Helsinki. All patients provided written informed consent.

### 2.2 Radiological evaluation

Positron emission tomography/CT imaging was assessed at baseline in all patients. Tumor evaluation with CT was performed every 6 weeks in the first year of ICI treatment, thereafter every 12 weeks until disease progression. RECIST v1.1 criteria were used to assess tumor response. CtDNA dynamics were used to predict radiological response and DCB. Progressive disease (PD) is defined as an increase in tumor volume of > 20% or appearance of new lesions. Partial response (PR) is defined as a decrease in tumor volume of > 30%; complete response (CR) as response showing that all lesions (both target and nontarget) are less than 10mm in the long axis (except lymph nodes which have to be smaller than 10mm in short axis). SD is attributed if neither the criteria for PD, PR or CR are met.

### 2.3 Plasma collection and ccfDNA extraction

Blood samples were available in either vacutainer EDTA tubes (vacutainer #367525, Becton Dickinson, Franklin Lakes, NJ, USA; until December 2017) or cell‐free DNA blood Streck collection tubes (BCTs; Streck, Omaha, NE, USA), since January 2018. Processing of cell‐free plasma and ccfDNA extraction was according to standard operating procedure as reported previously [28, 29]. In short, EDTA blood samples were processed within 4 h and Streck samples within 24 h. Subsequent processing consisted of a slow (for EDTA: 820 ***g***, 10 min, 4 °C; for Streck: 1600 ***g***, 10 min, 20 °C) and subsequent fast (16 000 ***g***, 10 min, 4 °C) centrifugation step. Plasma was stored as 1 mL aliquots at −80 °C until ccfDNA extraction. CcfDNA was extracted from ~ 2 mL plasma using the QIAamp Circulating Nucleic Acid kit (Qiagen, Hilden, Germany) according to the manufacturer's recommendations and as reported previously [28]. CcfDNA was eluted in 52 µL of AVE buffer and its concentration was measured by Qubit™ dsDNA HS assay kit on a Qubit™ 2.0 fluorometer (Thermo Fisher Scientific, Waltham, MA, USA).

To determine the most appropriate timepoint after start ICI therapy to measure changes in ctDNA levels, a subset of 27 patients was first selected from whom plasma was stored of several timepoints between baseline and disease progression, as well as four patients who displayed rapid disease progression (within 6 weeks; Table S1). For this subset, 164 plasma samples were collected with on average 6 (2–12) samples per patient. After the appropriate timepoint of follow‐up was established, all 100 patients were analyzed at baseline (t_0_) and at 4‐ to 6‐week follow‐up (t_1_).

### 2.4 Tumor specimen handling and tissue NGS

As routine workup of suspected lung cancer, tumor tissue was obtained by a bronchoscopy, transthoracic biopsy or an endoscopic ultrasound procedure (endobronchial ultrasound/endoscopic ultrasound). Tissue samples were processed and diagnosed following routine pathology procedures. Following Dutch guidelines, FFPE‐pretreatment tissue samples of all adenocarcinomas from patients with metastasized NSCLC were subjected to sequence analysis by targeted NGS for mutations in relevant predictive markers including *EGFR*, *BRAF*, *KRAS*, *PIK3CA*, erb‐b2 receptor tyrosine kinase 2 and *MET* [30] in the NEN‐EN‐ISO15189‐accredited laboratory for molecular pathology at the UMCG as reported previously [20, 31]. Molecular results are reported in the Dutch nationwide pathology registry (PALGA). For this study, lung adenocarcinoma patients were selected with a somatic mutation for which no targetable drugs were available and therefore were treated with ICI (see Table 1 for overview of mutations). Out of 22 patients with non‐*KRAS* mutations, 11 patients with a targetable mutation (e.g., *BRAF* V600E, *EGFR* L858R, or *EGFR* T790M) were included following progression on tyrosine kinase inhibitors (TKIs) or as a last resort treatment. PD‐L1 expression was detected with the Ventana PD‐L1 (SP263) Assay (RTU, conformité Européene‐*in vitro* diagnostic) on a Ventana Benchmark Ultra immunostainer on pretreatment tissue biopsies. Staining was scored by an experienced pulmonary pathologist (WT) according to international classification criteria and reported as tumor proportion score (TPS) for 87 patients [32].

**Table 1:** Clinical and pathological characteristics. N/A, not available.

Patients	100
Median age	66 (29–85)
Sex	
Male	53 (53%)
Female	47 (47%)
ECOG PS	
0	42 (42%)
1	49 (49%)
2	7 (7%)
3	2 (2%)
Smoking status	
Current	39 (39%)
Former	58 (58%)
Never	3 (3%)
Immunotherapy	
Atezolizumab	2 (2%)
Durvalumab	1 (1%)
Nivolumab	69 (69%)
Pembrolizumab	28 (28%)
Previous lines of (chemo)therapies	
0	25 (25%)
1	57 (57%)
2	12 (12%)
3	6 (6%)
*KRAS* mutations	78 (78%)
c.35G>C p.(G12A)	4 (4%)
c.34G>T p.(G12C)	37 (37%)
c.35G>A p.(G12D)	9 (9%)
c.34G>C p.(G12R)	1 (1%)
c.35G>T p.(G12V)	18 (18%)
c.37G>T p.(G13C)	1 (1%)
c.38G>A p.(G13D)	3 (3%)
c.183A>C p.(Q61H)	3 (3%)
c.181C>A p.(Q61K)	1 (1%)
c.182A>T p.(Q61L)	1 (1%)
Non‐*KRAS* mutations	22 (22%)
*BRAF* c.1397G>C p.(G466A)	1 (3%)
*BRAF* c.1397G>T p.(G466V)	2 (1%)
*BRAF* c.1406G>C p.(G469A)	3 (1%)
*BRAF* c.1406G>T p.(G469V)	1 (1%)
*BRAF* c.1799_1801del p.(V600_K601delinsE)	1 (1%)
*BRAF* c.1799T>A p.(V600E)	5 (5%)
*EGFR* c.2310_2311insGGC p.(D770_N771insG)	1 (1%)
*EGFR* c.2155G>A p.(G719S)	1 (1%)
*EGFR* c.2316_2321dup p.(H773_V774dup)	1 (1%)
*EGFR* c.2573T>G p.(L858R)	1 (1%)
*PIK3CA* c.1624G>A p.(E542K)	3 (5%)
*PIK3CA* c.1633G>A p.(E545K)	2 (3%)
PD‐L1 TPS	
< 1%	34 (34%)
1–49%	17 (17%)
≥ 50%	35 (35%)
N/A	14 (14%)

### 2.5 Quantitative ctDNA analysis

For each patient, a tumor‐specific ddPCR assay using nontargetable (driver) mutations present in the pretreatment biopsy was selected in order to detect and quantify the tumor‐specific mutations in ccfDNA (Table S2). DdPCR analysis was performed as reported previously [20, 23, 28]. In short, ccfDNA (median 5.4 ng, 1.3–61 ng) was emulsified into 10.000–20.000 droplets by the QX200™ droplet generator (Bio‐Rad Laboratories, Pleasanton, CA, USA) and amplified with ddPCR™ supermix (Bio‐Rad) and the primers and probes (Table S2) into a final volume of 20 µL. Mutant (FAM‐labeled) or wild‐type (HEX‐labeled) fluorescent quantitative signals were detected by the QX200™ platform (Bio‐Rad). DdPCR results were analyzed with QuantaSoft™ analytical software (Bio‐Rad). Droplet counts were used to calculate the number of mutant copies per mL of plasma. The variant allelic frequency was determined by QuantaSoft™ Analysis Pro. Samples were regarded as positive if ≥ 3 mutant droplets were detected and negative if < 3 mutant droplets with at least 330 total positive (wild‐type and mutant) droplets were detected (ensuring an analytical sensitivity < 1%). Because previous assessments of the precision of the ddPCR tests that are used in this study revealed a 30% technical variance [23], we set the minimum threshold at 30% and we only consider changes in mutant ctDNA levels greater than 30% as a true increase or decrease. In addition, we evaluated more stringent thresholds of 40% and 50% that were previously reported to be informative [12, 33, 34]. To confirm the changes in ctDNA levels detected with ddPCR, a fully automated real‐time PCR Idylla™ ctKRAS Mutation Assay (Biocartis, Mechelen, Belgium) was performed as reported previously [35, 36]. All analyses included mutation‐positive, wild‐type, and no template controls. All standard precautions were taken to avoid contamination of amplification products using separate laboratories for pre‐ and post‐PCR handling. Clinical and laboratory test outcomes were independently added into the database.

### 2.6 Statistical analysis

Descriptive statistics were used for patient and tumor characteristics. PFS and OS were defined as the period between the date of start of ICI to the date of PD or date of death, respectively. Data were censored at the date of last follow‐up in absence of an event. Kaplan–Meier survival data were stratified for mutant ctDNA data and compared with the log‐rank test. To compare ctDNA dynamics with PD‐L1 TPS, Kaplan–Meier curves were stratified according to the PD‐L1 TPS. Radiological reports and liquid biopsy test results were assessed independently. Correlation between the *KRAS* G12/13 screening ddPCR assay and Idylla™ ctKRAS Mutation Assay results was determined using Pearson's correlation coefficient and agreement was performed using Cohen's κ. Differences in the rate of DCB were assessed with a Mann–Whitney *U* test. graphpad prism 8.4.2 (GraphPad Software, San Diego, CA, USA) or spss version 25 software (IBM SPSS Statistics, Armonk, NY, USA) were used for all statistical analysis, wherein a *P*‐value < 0.05 was considered significant.

## 3 Results

### 3.1 Patient characteristics

Next‐generation sequencing analysis of the pretreatment FFPE tissue biopsies identified 78 tumor samples with mutations in *KRAS* (78%) and 22 with a non‐*KRAS* mutation (22%). All clinical and pathological characteristics are summarized in Table 1. Most patients (*n* = 69) were treated with nivolumab 3 mg·kg^−1^ body weight intravenously every 2 weeks or pembrolizumab 200 mg (*n* = 28 patients) every 3 weeks intravenously (Table 1). In addition, two patients were treated with atezolizumab 1200 mg every 2 weeks and one patient with durvalumab 20 mg·kg^−1^ every 2 weeks. The median number of weeks from start ICI until tumor response was 6 weeks (2–55 weeks). Follow‐up CT imaging was not performed in eight patients (8%) as clinical PD already occurred prior to the first radiological evaluation. Sixty‐six patients (66%) had an early tumor response, defined by a tumor response according to RECIST v1.1 within 6 weeks after start ICI treatment. A late tumor response, defined by tumor response according to RECIST v1.1 after 12 weeks, was observed in 18 (18%). A DCB is defined by a clinical response with at least SD lasting ≥ 6 months as reported previously [8], which was achieved in 39 patients (39%).

### 3.2 Optimal timepoint to measure changes in ctDNA levels associated with durable tumor response

**Fig. 1:**
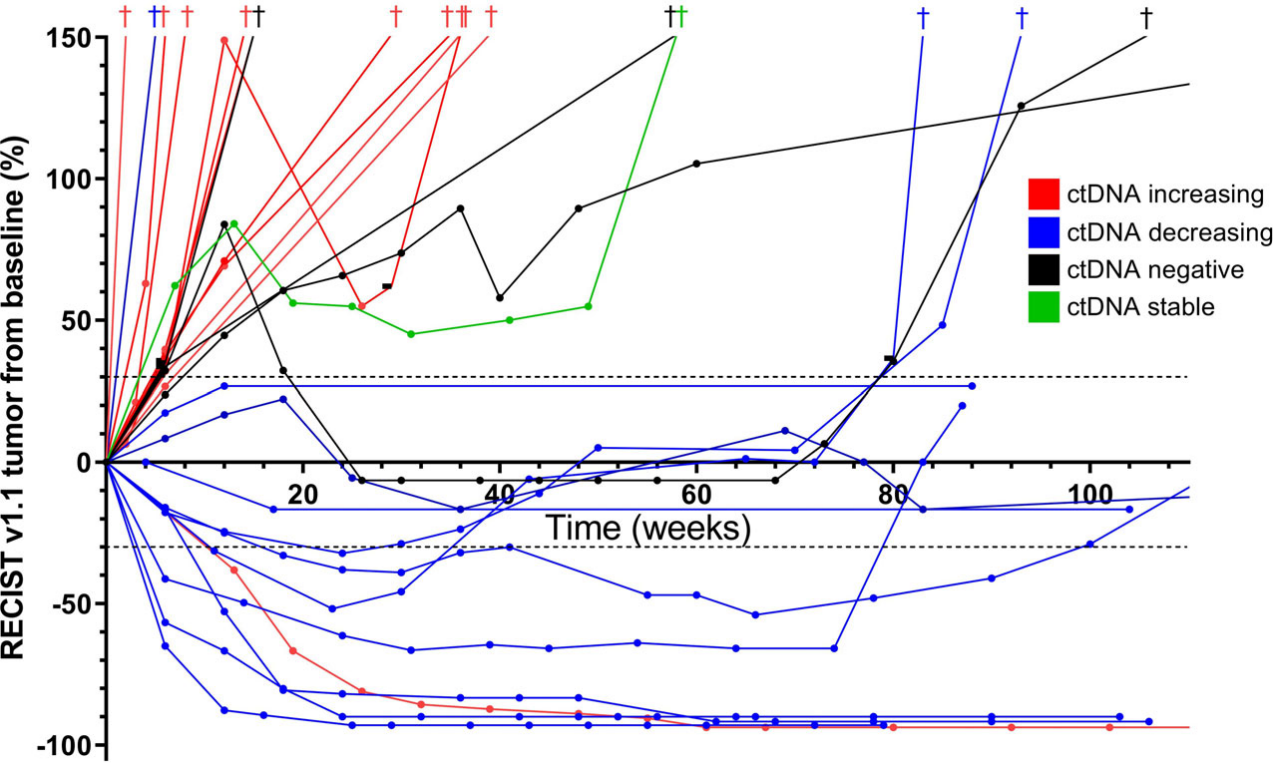
Spider plot analysis of radiological response according to the RECIST v1.1 criteria and changes in mutant ctDNA levels. CtDNA levels were determined by the difference in mutant copies per mL of plasma at baseline (t_0_) and 4–6 weeks after start of ICI treatment (t_1_). Dashed lines indicate a 20% increase and 30% decrease in tumor volume compared with baseline. The cross symbol indicates the patient's death at that point in time. One exceptional case is described in Fig. S3. CtDNA increasing, 30% more mutant copies at t_1_ compared with t_0_; ctDNA decreasing, 30% less mutant copies at t_1_ compared with t_0_; ctDNA‐negative, driver mutation in tissue not detected in plasma; ctDNA stable, observed change in mutant copies at t_1_ compared with t_0_ was ≤ 30%.

Twenty‐seven patients with a *KRAS* or *BRAF* (non‐V600E) mutation in the primary tumor from whom plasma was available at several timepoints during ICI treatment, predominantly at 1, 2, 4, and 6 weeks after initiation, were selected (Table S1) to determine the optimal timepoint to measure changes in ctDNA levels associated with therapy response effects. CcfDNA was analyzed to quantify mutant ctDNA copies. Tumor response patterns could be divided into five typical patterns for CR, PR, SD, PD, and ctDNA‐negative patients (see examples in Fig. S1A–E). The ctDNA patterns of all responding patients (*n* = 11) revealed an initial spike in ctDNA levels prior to a decrease in ctDNA levels (Fig. S2A). One exceptional case is discussed separately (Fig. S3). In samples at 4–6 weeks, most of the responders (70–89%) showed a > 30% decrease, while in most of the nonresponders (55–75%) ctDNA levels at 4–6 weeks increased (Fig. S2B). Spider plot analysis supported the predictive value of ctDNA analysis 4–6 weeks after start therapy (t_1_). Patients with increased, stable or nondetectable (considered as negative) levels of ctDNA demonstrated early disease progression, of whom 14/16 (88%) have deceased. The majority of patients with decreasing ctDNA demonstrated a response, of whom 10/11 (91%) were alive after at least 80 weeks (Fig. 1).

### 3.3 Validation of KRAS ddPCR analysis with Idylla ctKRAS

To confirm the levels of *KRAS*‐mutated ctDNA detected in cell‐free plasma using ddPCR analysis, 89 samples with sufficient plasma were also analyzed with the Idylla™ ctKRAS Mutation Assay as an independent plasma‐based test. Based on the number of mutant copies per mL plasma, ddPCR and Idylla revealed similar results (*r* ^2^ = 0.94, black line; *r* ^2^ = 0.64 omitting six cases with very high levels, blue line; Fig. S4). When comparing changes in *KRAS* mutant ctDNA levels between t_0_ and t_1,_ 13 of the 15 patients showed a similar association with clinical response represented by an almost perfect agreement when comparing ddPCR with Idylla (κ = 0.84). These data confirmed that quantitative ctDNA analysis using ddPCR reliably predicted changes in mutant ctDNA levels.

### 3.4 Changes in ctDNA levels as an early marker of durable clinical benefit

To validate the potential value of monitoring ctDNA levels, ddPCR analysis was performed on ccfDNA from 100 lung adenocarcinoma patients treated with mono‐immunotherapy. When ctDNA was detected at t_0_, a significant difference in the number of mutant copies per mL of plasma was observed between patients with no clinical response and patients who had a DCB (Fig. S5A). Patients with high mutant ctDNA levels at t_0_ showed a poorer PFS (*P* < 0.001) and OS (*P* < 0.0001) compared with low mutant copy levels (Fig. S5B,C). No ctDNA was detected at t_0_ in 31 patients (31%). CtDNA‐negative patients were represented both in 21 of the 63 nonresponders (33%) and 10 of 37 of durable responders (27%) (Fig. S5A, red dots).

**Fig. 2:**
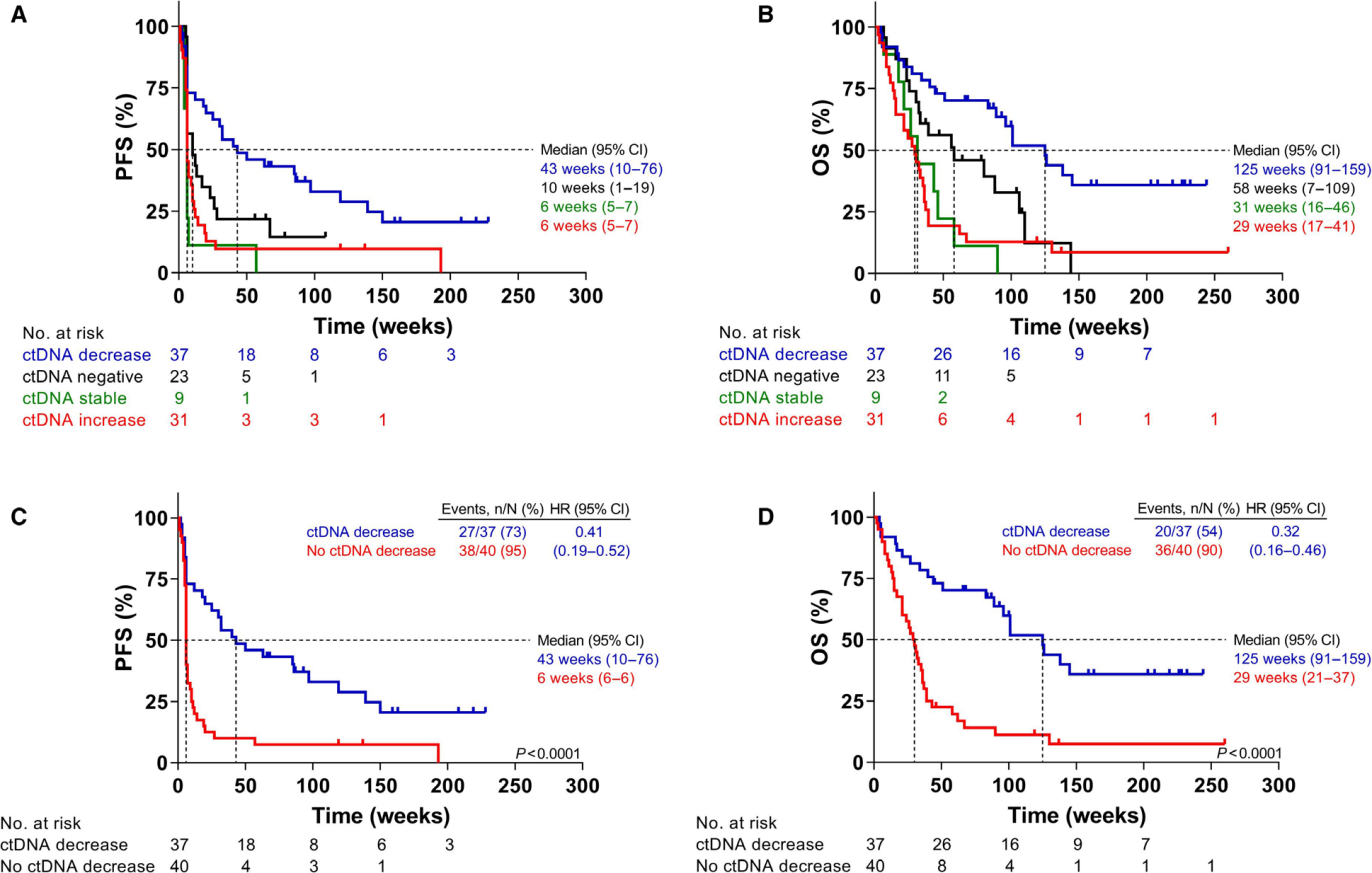
Tumor response related to changes in mutant ctDNA levels. Kaplan–Meier plot displaying the (A) PFS and (B) OS of patients with decreasing (blue), negative (black), stable (green), or increasing (red) ctDNA levels. (C) PFS and (D) OS of patients with decreasing ctDNA levels (blue), or no decrease in ctDNA (red). Log‐rank test, *P*‐values of < 0.05 are considered significant. CtDNA decreasing, 30% less mutant copies at t_1_ compared with t_0_; ctDNA‐negative, driver mutation in tissue not detected in plasma; ctDNA stable, observed change in mutant copies at t_1_ compared with t_0_ was ≤ 30%; ctDNA increasing, 30% more mutant copies at t_1_ compared with t_0_; no decrease in ctDNA, encompasses patients with ctDNA increase and ctDNA stable.

Patients with a decrease in ctDNA levels at t_1_ had the best median PFS and OS (Fig. 2A,B). Patients with both stable ctDNA (change at t_1_ compared with t_0_ ≤ 30%) or increased (> 30%) ctDNA levels showed similar poor responses. Therefore, patients with a ctDNA increase or ctDNA stable levels were grouped as no ctDNA decrease in subsequent analyses. Although 70% of patients without detectable ctDNA (16/23) showed early disease progression (within 6 months), they did perform better than patients with increasing or stable levels of ctDNA, but worse than those with a decrease in ctDNA was observed at t_1_ (Fig. 2B). Therefore, patients without detectable ctDNA were regarded as a separate group.

Analysis excluding ctDNA‐negative patients revealed that patients with decreasing mutant ctDNA levels had a significantly improved PFS (hazard ratio [HR]: 0.41 [0.19–0.52]; *P* < 0.0001) compared with patients who did not (no decrease in mutant ctDNA), resulting in a longer median PFS (43 vs 6 weeks; Fig. 2C) and OS (125 vs 29 weeks; HR: 0.32 [0.16–0.46]; *P* < 0.0001; Fig. 2D). Using a higher threshold of 50% for ctDNA response (Fig. S6) revealed comparable results as observed for 30% with only a slightly improved HRs for PFS and OS.

To exclude that the observed association between ctDNA levels and treatment response was due to the specific activity of *KRAS* mutations, the PFS and OS comparing presence (*n* = 78) or absence (*n* = 22) of *KRAS* mutations in the pretreatment tumor tissue were evaluated. This analysis revealed no significant difference in PFS and OS (Fig. S7).

### 3.5 PD‐L1 expression in pretreatment tissue biopsies and ctDNA dynamics

Progressive disease‐L1 expression data were available for 87 patients. Thirty‐five patients (40%) were PD‐L1 negative (TPS < 1%) and 52 (60%) had a PD‐L1 TPS ≥ 1% (of whom 35 with TPS ≥ 50%; Table 1). In this cohort, patients with a PD‐L1 TPS of ≥ 1% had a longer PFS (25 vs 6 weeks; HR: 0.46 [0.22–0.61]; *P* < 0.001) and OS (83 vs 32 weeks; HR: 0.57 [0.32–0.92]; *P* < 0.05) than PD‐L1 negative patients (Fig. S8).

**Fig. 3:**
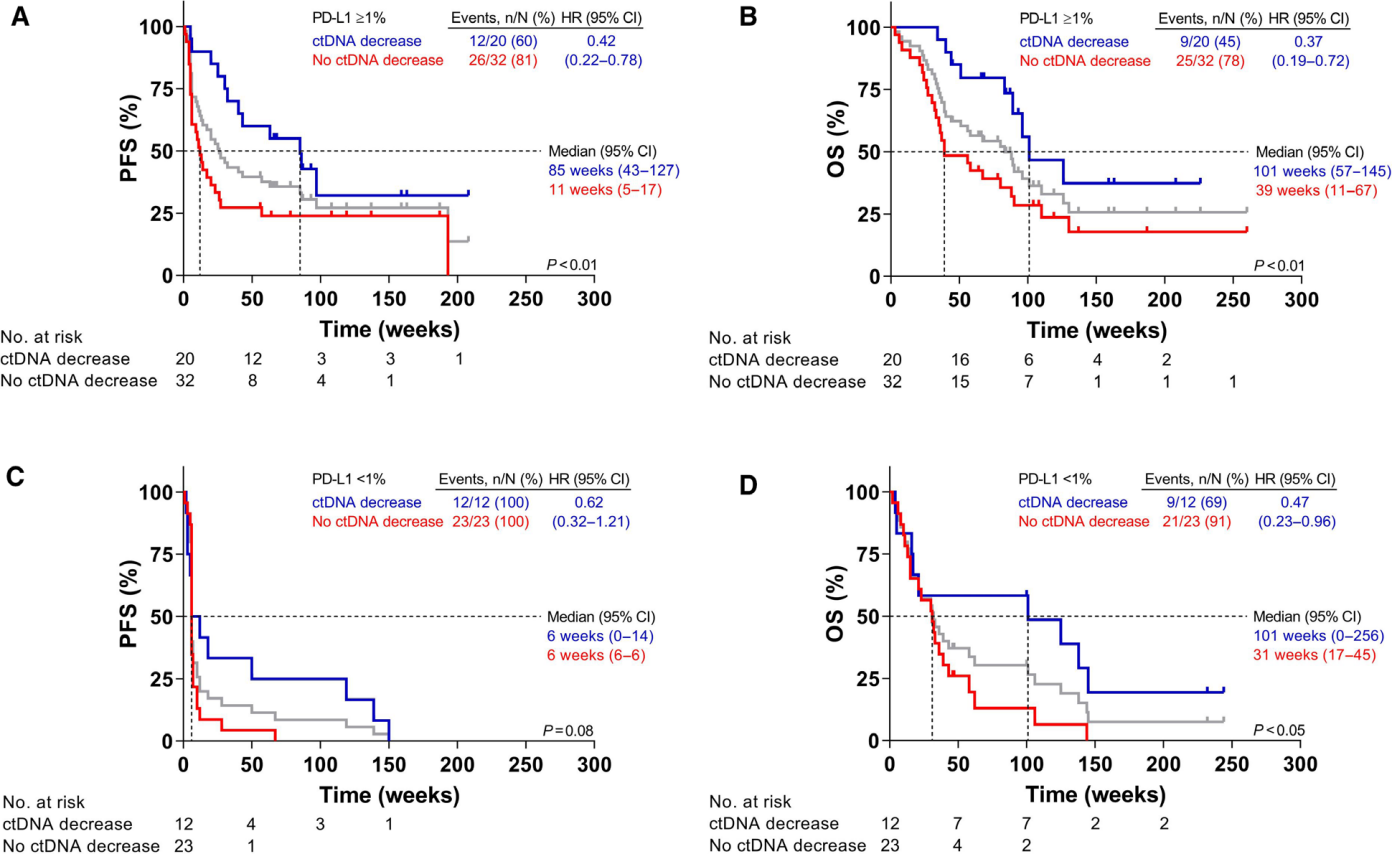
Tumor response related to change in mutant ctDNA levels and PD‐L1 TPS status. Kaplan–Meier plot displaying the (A, C) PFS and (B, D) OS of patients with a PD‐L1 TPS of ≥ 1% (A, B) and < 1% (C, D) with decreasing (blue), or increasing or stable (red) ctDNA levels. The gray lines represent the entire PD‐L1 cohort in the respective subgroups (not used in comparison of the different subgroups). Figure S9 shows the analysis of patients with decreasing, stable, increasing, and nondetectable ctDNA levels separately. Log‐rank test, *P*‐values of < 0.05 are considered significant. CtDNA decreasing, 30% less mutant copies at t_1_ compared with t_0_; no decrease in ctDNA, encompasses patients with ctDNA increase, ctDNA‐negative and ctDNA stable.

In patients with a PD‐L1 TPS of ≥ 1%, decreased ctDNA levels further improved both PFS (85 vs 11 weeks; HR: 0.42 [0.22–0.78]; *P* < 0.01) and OS (101 vs 39 weeks; HR: 0.37 [0.19–0.72]; *P* < 0.01; Fig. 3A,B; Fig. S9A,B). Interestingly, in a subset of PD‐L1‐negative patients (TPS of < 1%), decreased ctDNA levels were also associated with prolonged PFS and OS (Fig. 3C,D; Fig. S9C,D). The effect of a ctDNA decrease on PFS was stronger for patients with PD‐L1 expressing tumors compared with patients with PD‐L1‐negative tumors (HR: 0.40 [0.14–0.80], *P* < 0.05; data not shown).

## 4 Discussion

When a tumor‐derived molecular aberration is detected in plasma, this can potentially be used to monitor early tumor response to ICI. In the current study, we demonstrate the value of measuring ctDNA levels using ddPCR at baseline (t_0_) and follow‐up (4–6 weeks, t_1_) as a minimally invasive monitoring tool for response to ICI monotherapy. The group of patients who displayed a decrease in mutant copies had a longer PFS, OS, and DCB compared with those without decrease in ctDNA levels. Furthermore, patients who displayed a reduction in mutant tumor DNA in circulation and had a PD‐L1 expressing tumor demonstrated an even better PFS, OS, and DCB. The data indicate that the combination of PD‐L1 expression and reduction in ctDNA is a stronger monitoring tool for response to ICI than PD‐L1 expression or change in ctDNA alone.

Detection of tumor‐derived DNA in liquid biopsy has enabled assessment of mutation profiles in plasma of cancer patients at different stages of disease in a minimally invasive manner [37]. Recent studies advocate NGS of pretreatment plasma samples as the most appropriate approach to identify mutants for disease monitoring of virtually all patients. Subsequently, a selection of these mutations can be monitored in plasma over time. In current clinical practice however, high cost of plasma‐derived ccfDNA NGS for all patients is cost‐prohibitive. In contrast, it is currently common practice to perform molecular profiling on a tumor tissue biopsy with broader NGS mutation panels. Mutation profiling of tumor biopsies not only resulted in the identification of clinical‐relevant druggable targets, but also in tumor‐specific variants that may be detected in circulation. In the current study, the tumor‐informed ddPCR analysis of ctDNA has demonstrated promise as a cost‐effective monitoring tool.

We studied dynamics of mutant ctDNA levels prior to radiological evaluation in plasma using mutations that were detected in the pretreatment tissue biopsies as part of routine molecular diagnostics. In the first 2 weeks of treatment, a spike in ctDNA levels was observed in 61% of all patients with measurable ctDNA at baseline (14/23), and in 70% of patients who eventually demonstrated treatment response (Fig. S2). This transient spike in ctDNA was reported previously for *KRAS* and *EGFR* in NSCLC, probably reflecting tumor DNA release by death of tumor cells upon initiation of systemic treatment [11, 12, 38]. The strong increase in ctDNA within 2 weeks after start of therapy that was observed in 15 patients was not predictive for DCB (data not shown). Our analysis demonstrated that at least a 30% decrease in ctDNA levels at 4–6 weeks after initiation of treatment (t_1_) correlated with a longer PFS and OS in response to ICI treatment, as well as an increased rate of DCB (Table S3). A decrease in mutant ctDNA levels was associated with a superior median PFS (43 weeks, HR: 0.41 [0.19–0.52]) and OS (125 weeks, HR: 0.32 [0.16–0.46]) compared with that of combined patient group with increasing or stable ctDNA levels (PFS 6 weeks; OS 29 weeks). These results are comparable to three other studies with small cohorts of advanced NSCLC patient (respectively 14 [24], 34 [25], and 15 cases [26]) with nontargetable mutations detected in tumor biopsy treated with ICI. Despite that *KRAS*‐mutated tumors were associated with high PD‐L1 expression and consequently with increased tumor responses toward PD‐(L)1 inhibition [2, 39, 40, 41], no discrepancies between tumor harboring *KRAS* or other mutations were observed in our cohort.

In the current study, the median PFS of patients with a PD‐L1 TPS ≥ 1% is just 25 weeks. Further dividing PD‐L1 TPS in 1–49% and ≥ 50%, which is generally applied in current literature, did not reveal significant differences regarding PFS (*P* = 0.22) and OS (*P* = 0.15; data not shown). Combining independent biomarkers has previously shown to augment the predictive potential for DCB, as previously shown for plasma NGS with CD8^+^ cell levels [14]. When combining PD‐L1 immunohistochemistry in pretreatment tumor biopsies with changes in ctDNA levels, these changes did not correlate with PD‐L1 TPS, indicating that both markers are independent biomarkers (Fig. S10). In fact, combining changes in ctDNA with PD‐L1 TPS ≥ 1% showed an eightfold longer PFS and more than twofold longer OS in patients with a decrease in ctDNA levels compared with patients who did not show a > 30% decrease (Fig. 3). A subset of patients with a PD‐L1 TPS of < 1% with decreasing ctDNA levels seems to benefit from monotherapy as well (Table S4). Responders to immunotherapy in our study were observed both with high and low PD‐L1 tumors. The value of ctDNA decrease for monitoring treatment effect was independent of PD‐L1 expression. Reck *et al*. [3] also reported an improved response upon decrease in ctDNA at t_1_ in a patient cohort with PD‐L1 expression for first‐line ICI treatment using a cutoff of TPS ≥ 50%. In line with this observation, evaluation of patients with PD‐L1 TPS ≥ 50% and a decrease in ctDNA revealed even lower HRs (0.32 for PFS and 0.29 for OS; data not shown). However, in the current study 75% of patients was not treatment naïve. Patients who received previous lines of treatment generally show poorer response and survival times to ICI [4]. Despite the low number of patients in this study, this underscores the strong monitoring potential of change in ctDNA in combination with or without PD‐L1 expression and warrants further prospective evaluation. The sensitivity of this combination monitoring tool might further be augmented by addition of other potentially predictive biomarkers such as the immunoscore, immune infiltration, cytokine signatures (e.g., interferon gamma, transforming growth factor beta), and somatic copy number alterations [42, 43, 44].

In patients with known driver mutations, these mutations are not retrieved in approximately 30% of matched cell‐free plasma in various malignancies [45]. In line with these observations, in 31% of the included patients with metastasized disease the mutation detected in the pretreatment tumor biopsy was not detected in the corresponding ccfDNA sample at t_0_. No ctDNA was detected in 23% of the patients at both timepoints. Although the majority of patients without detectable ctDNA did not display a tumor response to treatment, their tumors seemed to have a more indolent course than those who did have specific ctDNA. This group of patients did have early PD in general, but OS was markedly better than for the ctDNA group showing stable levels or an increase at t_1_. The cause of absence of ctDNA in these plasma remains uncertain and proposed mechanisms include nonshedding tumors, increased clearance, shorter half‐life, lack of sufficient analytical sensitivity, and stage of disease [37, 45].

To monitor tumor response in ccfDNA using mutation‐specific ddPCR analysis, sequencing of pretreatment tumor tissue is required to select a tumor‐specific target. In 50% of advanced‐stage NSCLC targetable (~ 20%) or nontargetable *KRAS* (~ 30%) driver mutations are detected with current commonly‐used diagnostic NGS approaches [31, 45]. However, mutations detected in the tumor may not always be present in plasma. Broadening routine clinical tissue NGS panels, for example, with the frequently mutated *TP53* and *STK11* genes, will increase the number of patients who can be effectively monitored for tumor response using plasma ccfDNA with single‐gene approaches such as ddPCR. In this study, five patients with tumors containing multiple mutations at least one of these mutations could not be detected in the plasma. Selection of a mutation for monitoring purposes in plasma might lead to inconsistent results (Table S5). As such, several studies in lung cancer advocate the use of NGS analysis with a broad panel of markers on baseline plasma samples instead of a single selected marker. Targeting multiple mutations simultaneously also elevates the sensitivity of detecting ctDNA [12, 46]. Indeed, the number of ctDNA‐negative patients when using NGS approaches is substantially lower (4–8%) than was observed with our single variant assay [14, 47]. Studies that used an NGS approach to monitor ctDNA in response to ICI therapy demonstrate a correlation between ctDNA dynamics and response similar to our findings [12, 13, 48]. Recently, three studies comprising larger cohorts of various malignancies including NSCLC treated with ICI reported on the association between serial ctDNA NGS testing and PFS, OS, clinical response, and clinical benefit [14, 15, 16]. However, in current clinical practice, NGS approaches on ccfDNA are not yet cost‐effective for monitoring the course of treatment longitudinally. Single‐target ddPCR analysis therefore provides a cost‐effective alternative when the ctDNA target is detectable in the circulation. Longitudinal monitoring of a single tumor‐derived variant beyond the currently proposed interval might assist in early detection of disease progression and its clinical applicability, probably in combination with multiple available biomarkers, should be investigated in future (prospective) studies. Besides, as ccfDNA is shed into circulation from various tissues, DNA fragments from hematopoietic and germline origin are prone to affect analytical results with NGS, as well as inconsistent preanalytical handling and sample processing [23, 49, 50, 51]. Although the majority of clonal hematopoietic variants occur in nontargetable genes, these variants are also identified in targetable genes such as *KRAS*, *BRAF*, and *PIK3CA* as well [16]. Deep sequencing of plasma may therefore identify more mutations, but these might not all be derived from the tumor. To this extent, parallel sequencing of a patient‐matched bloodborne reference material, for example, white blood cells, is of importance [50], further increasing the costs for routine clinical practice. Therefore, monitoring ctDNA with a ddPCR assay is as sensitive as NGS to monitor therapy response but in a cost‐effective manner. However, ddPCR is only informative when tumor‐derived DNA is present in circulation.

## 5 Conclusion

Altogether, decreasing mutant copies estimated with ddPCR were associated with longer PFS and OS compared with patients displaying increased or stable ctDNA levels. CtDNA dynamics in combination with PD‐L1 status is a promising cost‐effective approach to monitor DCB, PFS, and OS in patients treated with ICI. Measuring a single tumor‐derived molecular aberration, when retrieved in the circulation, improves the early recognition of DCB and can assist in treatment decision making.

## Conflict of interest

NR is employed by the Dutch Cancer Society (KWF, Alpe D'HuZes research grant RUG 2013‐6355). The other authors declare that the research was conducted in the absence of any commercial or financial relationships that could be construed as a potential conflict of interest. LCK received research grants from AstraZeneca, nanoString, Invitae‐ArcherDX, Bayer, and Roche, and an honorarium as a consultant or speaker for Pfizer, Novartis, AstraZeneca, BMS, nanoString, and Asuragen. ES has performed lectures for Bio‐Rad, Novartis, Roche, Biocartis, Illumina, Pfizer, AstraZeneca, and Agena Bioscience, is consultant in advisory boards for AstraZeneca, Roche, Pfizer, Novartis, Bayer, Lilly, BMS, Amgen, Biocartis, Illumina, Agena Bioscience, and MSD/Merck, and received research grants from Pfizer, Biocartis, Invitae‐ArcherDX, AstraZeneca, Agena Bioscience, BMS, Bio‐Rad, Roche, Boehringer Ingelheim.

## Author contributions

PL, BH, TJNH, and ES were involved in the conception and design of the study. PL, AM, MLAA, and NR performed the laboratory experiments. PL, BH, AE, WT, HJMG, LCK, TJNH, and ES were responsible for the patient data collection and data curation. PL, BH, LCK, TJNH, and ES prepared the original draft. All authors contributed to data interpretation, critically revised the article, and approved the final version.

### Peer Review

The peer review history for this article is available at https://publons.com/publon/10.1002/1878‐0261.13090.

## Supporting information


**Fig. S1**. Patterns of response combining mutant ctDNA levels and tumor volume using CT scanning.
**Fig. S2**. Determination of most appropriate timepoints to optimally detect changes in ctDNA levels related to treatment response.
**Fig. S3**. Evaluation of case B‐003.
**Fig. S4**. Correlation of the mutant copies per mL of plasma as determined with ddPCR and Idylla™ ctKRAS Mutation Assay.
**Fig. S5**. Mutant ctDNA levels at baseline.
**Fig. S6**. PFS and OS at different cut‐offs to determine ctDNA decrease.
**Fig. S7**. PFS and OS is irrespective of *KRAS* mutations.
**Fig. S8**. Clinical response related to PD‐L1 expression.
**Fig. S9**. Elaborate analysis of radiological response related to PD‐L1 expression.
**Fig. S10**. No correlation between change in ctDNA levels and PD‐L1 TPS.
**Table S1**. Overview of subgroup of 27 patients to determine the most appropriate timepoint after start ICI therapy to measure clinically relevant changes in ctDNA.
**Table S2**. Assays used for ddPCR analysis.
**Table S3**. CtDNA dynamics and clinical response.
**Table S4**. CtDNA dynamics and PD‐L1 TPS score.
**Table S5**. Patients with multiple targetable mutations.

## Data Availability

Supporting anonymized ddPCR and clinical response data are available for sharing upon reasonable request.
