# Erratum to: Circulating tumor DNA as a biomarker for monitoring early treatment responses of patients with advanced lung adenocarcinoma receiving immune checkpoint inhibitors

**DOI:** 10.1002/1878-0261.13139

**Published:** 2022-01-05

The following error appeared in Section 3.5 in Ref. [1]. Instead of ‘Progressive disease‐L1 expression data were available for 87 patients’, the text should read ‘PD‐L1 expression data were available for 87 patients’.

We apologize for this error.
