# Supplementary material for: Circulating tumor DNA as a biomarker for monitoring early treatment responses of patients with advanced lung adenocarcinoma receiving immune checkpoint inhibitors
Source: Mol Oncol. 2021 Sep 25;15(11):2910–22. doi: 10.1002/1878-0261.13090 (PMC8564646; doi:10.1002/1878-0261.13090)
Supplement: Supplementary file 1 — Fig. S1. Patterns of response combining mutant ctDNA levels and tumor volume using CT scanning. Fig. S2. Determination of most appropriate timepoints to optimally detect changes in ctDNA levels related to treatment response. Fig. S3. Evaluation of case B‐003. Fig. S4. Correlation of the mutant copies per mL of plasma as determined with ddPCR and Idylla™ ctKRAS Mutation Assay. Fig. S5. Mutant ctDNA levels at baseline. Fig. S6. PFS and OS at different cut‐offs to determine ctDNA decrease. Fig. S7. PFS and OS is irrespective of KRAS mutations. Fig. S8. Clinical response related to PD‐L1 expression. Fig. S9. Elaborate analysis of radiological response related to PD‐L1 expression. Fig. S10. No correlation between change in ctDNA levels and PD‐L1 TPS. Table S1. Overview of subgroup of 27 patients to determine the most appropriate timepoint after start ICI therapy to measure clinically relevant changes in ctDNA. Table S2. Assays used for ddPCR analysis. Table S3. CtDNA dynamics and clinical response. Table S4. CtDNA dynamics and PD‐L1 TPS score. Table S5. Patients with multiple targetable mutations. [file MOL2-15-2910-s001.docx]

**Supplementary material**

**Supplementary Tables**

Supplementary Table 1. Overview of subgroup of 27 patients to determine the most appropriate timepoint after start ICI therapy to measure clinically relevant changes in ctDNA.

| **Patient ID** | **Tumor mutation** | **RECIST v1.1 response** | **Timepoints available plasma samples**  **(weeks after start of treatment)** |
| --- | --- | --- | --- |
| B-001 | *KRAS* c.38G>A p.(G13D) | PD (non-response) | 0, 3* |
| B-002 | *BRAF* c.1406G>C p.(G469A) | PR (response) | 0, 2, 4, 6, 12, 24, 36, 48, 60, 72, 84, 96 |
| B-003 | *KRAS* c.34G>T p.(G12C) | PR (response) | 0, 1, 2, 13, 24, 34, 48, 61, 72, 84, 96, 108 |
| B-004 | *KRAS* c.35G>C p.(G12A) | SD (non-response) | 0, 1, 2, 4, 6, 12 |
| B-005 | *KRAS* c.35G>A p.(G12D) | PR (response) | 0, 1, 5, 9, 13, 36 |
| B-006 | *KRAS* c.34G>T p.(G12C) | PR (response) | 0, 1, 2, 4, 6, 24, 36, 48, 60 |
| B-007 | *KRAS* c.183A>C p.(Q61H) | PD (non-response) | 0, 1, 2, 4 |
| B-008 | *KRAS* c.34G>T p.(G12C) | PD (non-response) | 0, 2, 4* |
| B-009 | *KRAS* c.35G>A p.(G12D) | PD (non-response) | 0, 1, 2, 4, 6 |
| B-010 | *KRAS* c.35G>C p.(G12A) | PD (non-response) | 0, 2, 4, 6 |
| B-011 | *BRAF* c.1406G>C p.(G469A) | SD (non-response) | 0, 1, 2, 4, 6, 12 |
| B-012 | *KRAS* c.34G>T p.(G12C) | PD (non-response) | 0, 1, 4, 6 |
| B-013 | *KRAS* c.35G>A p.(G12D) | PR (response) | 0, 1, 2, 4, 6, 12 |
| B-014 | *KRAS* c.35G>A p.(G12D) | PR (response) | 0, 1, 2, 4, 6, 12 |
| B-015 | *KRAS* c.35G>T p.(G12V) | SD (non-response) | 0, 1, 2, 4, 6, 12, 24, 36, 60 |
| B-016 | *KRAS* c.34G>T p.(G12C) | SD (non-response) | 0, 1, 2, 4, 6, 12, 24, 36, 48, 74 |
| B-017 | *KRAS* c.34G>T p.(G12C) | PD (non-response) | 0, 1, 6, 70 |
| B-018 | *KRAS* c.34G>T p.(G12C) | SD (non-response) | 0, 1, 2, 4, 6, 12 |
| B-019 | *KRAS* c.35G>T p.(G12V) | PD (non-response) | 0, 1, 2, 4, 6 |
| B-020 | *KRAS* c.34G>T p.(G12C) | PD (non-response) | 0, 1* |
| B-021 | *KRAS* c.34G>T p.(G12C) | CR (response) | 0, 1, 2, 4, 6, 12, 23, 37, 46, 58 |
| B-022 | *KRAS* c.34G>T p.(G12C) | PD (non-response) | 0, 1, 2, 4, 13 |
| B-023 | *KRAS* c.35G>T p.(G12V) | PD (non-response) | 0, 5* |
| B-024 | *KRAS* c.34G>T p.(G12C) | PR (response) | 0, 1, 2, 4 |
| B-025 | *KRAS* c.34G>T p.(G12C) | CR (response) | 0, 1, 2, 4, 6, 8, 12, 24, 36 |
| B-026 | *KRAS* c.35G>T p.(G12V) | PR (response) | 0, 1, 2, 7, 24 |
| B-027 | *KRAS* c.35G>T p.(G12V) | PD (non-response) | 0, 1, 2, 4, 6 |

*Patients with rapid disease progression (within 6 weeks). RECIST, Response Evaluation Criteria in Solid Tumors; PD, progressive disease; SD stable disease; PR, partial response; CR, complete response.

Supplementary Table 2. Assays used for ddPCR analysis.

| **Bio-Rad assay name** | **Bio-Rad assay ID** | **Targeted nucleotide sequence** |
| --- | --- | --- |
| *BRAF* p.G466A | dHsaMDS389209582 | NM_004333: *BRAF* c.1397G>A |
| *BRAF* p.G466V | dHsaMDS2510966 | NM_004333: *BRAF* c.1397G>T |
| *BRAF* p.G469A | dHsaMDV2516932 | NM_004333: *BRAF* c.1406G>C |
| *BRAF* p.G469V | dHsaMDS747800353 | NM_004333: *BRAF* c.1406G>T |
| *BRAF* p.V600E | dHsaMDV2010027 | NM_004333: *BRAF* c.1799T>A |
| *BRAF* p.V600_K601>E | dHsaMDS890722866 | NM_004333: *BRAF* c.1799_1801delTGA |
| *EGFR* p.D770_N771insG | dHsaMDS625148063 | NM_005228: *EGFR* D770_N771insG |
| *EGFR* p.G719S | dHsaMDV2010041 | NM_005228: *EGFR* c.2155G>A |
| *EGFR* p.V774_C775insHV c.2315_2316insCCACGT* | dHsaMDS712821910 | NM_005228: *EGFR* c.2315_2316insCCACGT |
| *EGFR* p.L858R c.2573T>G | dHsaMDV2010021 | NM_005228: *EGFR* c.2573T>G |
| *EGFR* p.T790M | dHsaMDV2010019 | NM_005228: *EGFR* c.2369C>T |
| ddPCR *KRAS* G12/G13 Screening Kit | 1863506 | † |
| ddPCR *KRAS* Q61 Screening Kit | 12001626 | ‡ |
| *PIK3CA* p.E542K | dHsaMDV2010073 | NM_006218: *PIK3CA* c.1624G>A |
| *PIK3CA* p.E545K | dHsaMDV2010075 | NM_006218: *PIK3CA* c.1633G>A |

Assay IDs are displayed as provided by Bio-Rad Laboratories Inc. *Annotation of the *EGFR* p.V774_C775insHV assay according human genome variation society (HGVS) is *EGFR* p.(H773_V774dup). †The ddPCR *KRAS* G12/G13 Screening Kit was used to screen cases with a *KRAS* c.35G>C p.(G12A), c.34G>T p.(G12C), c.35G>A p.(G12D), c.34G>C p.(G12R), c.34G>A p.(G12S), c.35G>T p.(G12V) or c.38G>A p.(G13D) mutation. ‡The ddPCR *KRAS* Q61 Screening Kit was used to screen cases with a *KRAS* c.181C>A p.(Q61K), c.182A>T p.(Q61L), c.182A>G p.(Q61R), c.183A>T p.(Q61H) or c.183A>C p.(Q61H) mutation.

Supplementary Table 3. CtDNA dynamics and clinical response.

|  | PD | SD | PR | CR | NCR (<6 months) | DCB (≥6 months) |
| --- | --- | --- | --- | --- | --- | --- |
| **ctDNA decrease** | 10 (27%) | 5 (14%) | 16 (43%) | 6 (16%) | 14 (38%) | 23 (62%)^*^ |
| **No decrease in ctDNA** | 30 (75%) | 4 (10%) | 6 (15%) | 0 (0%) | 35 (88%) | 5 (12%) |
| **ctDNA negative** | 12 (52%) | 8 (35%) | 2 (9%) | 1 (4%) | 16 (70%) | 7 (30%) |

62% of the patients with decreased mutant copies display a DCB, as opposed to 12% of the patients with increasing or stable ctDNA levels (^*^*P*=0.0001, Mann-Whitney U test comparing PFS of patients with ctDNA decrease with no decrease in ctDNA. Although many patients without detectable mutant ctDNA levels both at t_0_ and t­_1_ (n=23) demonstrated early disease progression, 30% achieved a DCB. PD, progressive disease; SD stable disease; PR, partial response; CR, complete response; NCR, no clinical response; DCB, durable clinical benefit.

Supplementary Table 4. CtDNA dynamics and PD-L1 TPS score.

|  |  | PD | SD | PR | CR | NCR (<6 months) | DCB (≥6 months) |
| --- | --- | --- | --- | --- | --- | --- | --- |
| **TPS ≥1%** | **ctDNA decrease** | 2 (10%) | 1 (5%) | 12 (60%) | 5 (25%) | 4 (20%) | 16 (80%)^*^ |
|  | **No decrease in ctDNA** | 13 (59%) | 3 (14%) | 6 (27%) | 0 (0%) | 17 (77%) | 5 (23%) |
|  | **ctDNA negative** | 3 (30%) | 4 (40%) | 2 (20%) | 1 (10%) | 4 (40%) | 6 (60%) |
| **TPS <1%** | **ctDNA decrease** | 6 (46%) | 4 (31%) | 2 (23%) | 0 (0%) | 8 (62%) | 5 (38%)^†^ |
|  | **No decrease in ctDNA** | 15 (100%) | 0 (0%) | 0 (0%) | 0 (0%) | 15 (100%) | 0 (0%) |
|  | **ctDNA negative** | 5 (63%) | 3 (37%) | 0 (0%) | 0 (0%) | 5 (63%) | 3 (38%) |

Of the patients with decreasing mutant copies, 80% achieved a DCB, while 77% of patients displaying stable or ctDNA increase did not respond to treatment (^*^*P*<0.001, Mann-Whitney U test comparing PFS of patients with ctDNA decrease with no decrease in ctDNA). No significant difference in response rate was observed for patients with a PD-L1 TPS <1% (^†^*P*=0.31, Mann-Whitney U test comparing PFS of patients with ctDNA decrease with no decrease in ctDNA). TPS, PD-L1 Tumor Proportion Score; PD, progressive disease; SD stable disease; PR, partial response; CR, complete response; NCR, no clinical response; DCB, durable clinical benefit.

Supplementary Table 5. Patients with multiple targetable mutations.

|  | **Tissue** | | **Plasma mutant copies/mL** | | | **Radiology** |
| --- | --- | --- | --- | --- | --- | --- |
| **Patient** | **Mutation** | **VAF (%)** | **t_0_** | **t_1_** | **ctDNA change** | **RECIST v1.1** |
| **B-026** | *KRAS* c.35G>T p.(G12V) | 39% | 49.3 | 0.0 | Decrease | PR |
|  | *PIK3CA* c.1624G>A p.(E542K) | 12% | 0.0 | 0.0 | Negative |  |
| **B-032** | *EGFR* c.2573T>G p.(L858R) | 58% | 407 | 178 | Decrease | PR |
|  | *EGFR* c.2369C>T p.(T790M) | 3% | 0 | 0.0 | Negative |  |
| **B-065** | *KRAS* c.34G>T p.(G12C) | 11% | 727 | 632 | Stable | PD |
|  | *BRAF* c.1397G>T p.(G466V) | 10% | 0.0 | 0.0 | Negative |  |
| **B-072** | *PIK3CA* c.1633G>A p.(E545K) | 8% | 0.0 | 0.0 | Negative | SD |
|  | *PIK3CA* c.1624G>A p.(E542K) | 8% | 0.0 | 0.0 | Negative |  |
| **B-098** | *BRAF* c.1397G>T p.(G466V) | 51% | 55.4 | 0.0 | Decrease | PR |
|  | *BRAF* c.1799T>A p.(V600E) | 5% | 0.0 | 0.0 | Negative |  |

VAF, variant allelic frequency; mutant copies/mL, mutant copies per mL of plasma. RECIST, Response Evaluation Criteria in Solid Tumors; PD, progressive disease; SD stable disease; PR, partial response.

**Supplementary Figures**

**

** Supplementary Figure 1. Patterns of response combining mutant ctDNA levels and tumor volume using CT scanning. Display of the radiological response compared to baseline (t_0_, red) and changes in ctDNA levels (green) during treatment. Red dots and green circles represent timepoints that respectively CT-scanning was performed or plasma sample was drawn. Dashed lines indicate a 20% increase and 30% decrease in tumor volume compared to baseline. The bar on top shows which treatment the patients received over time, either immune checkpoint inhibitors (ICI, blue), chemotherapy (chemo, orange), targeted therapy (targeted, yellow) or no treatment (gray). Representative patterns are shown for patients displaying a (A) complete response (CR), (B) partial response (PR), (C) stable disease (SD), and (D) progressive disease (PD). (E) Most ctDNA negative patients showed progressive disease in an early stage, however did survive for a long time.


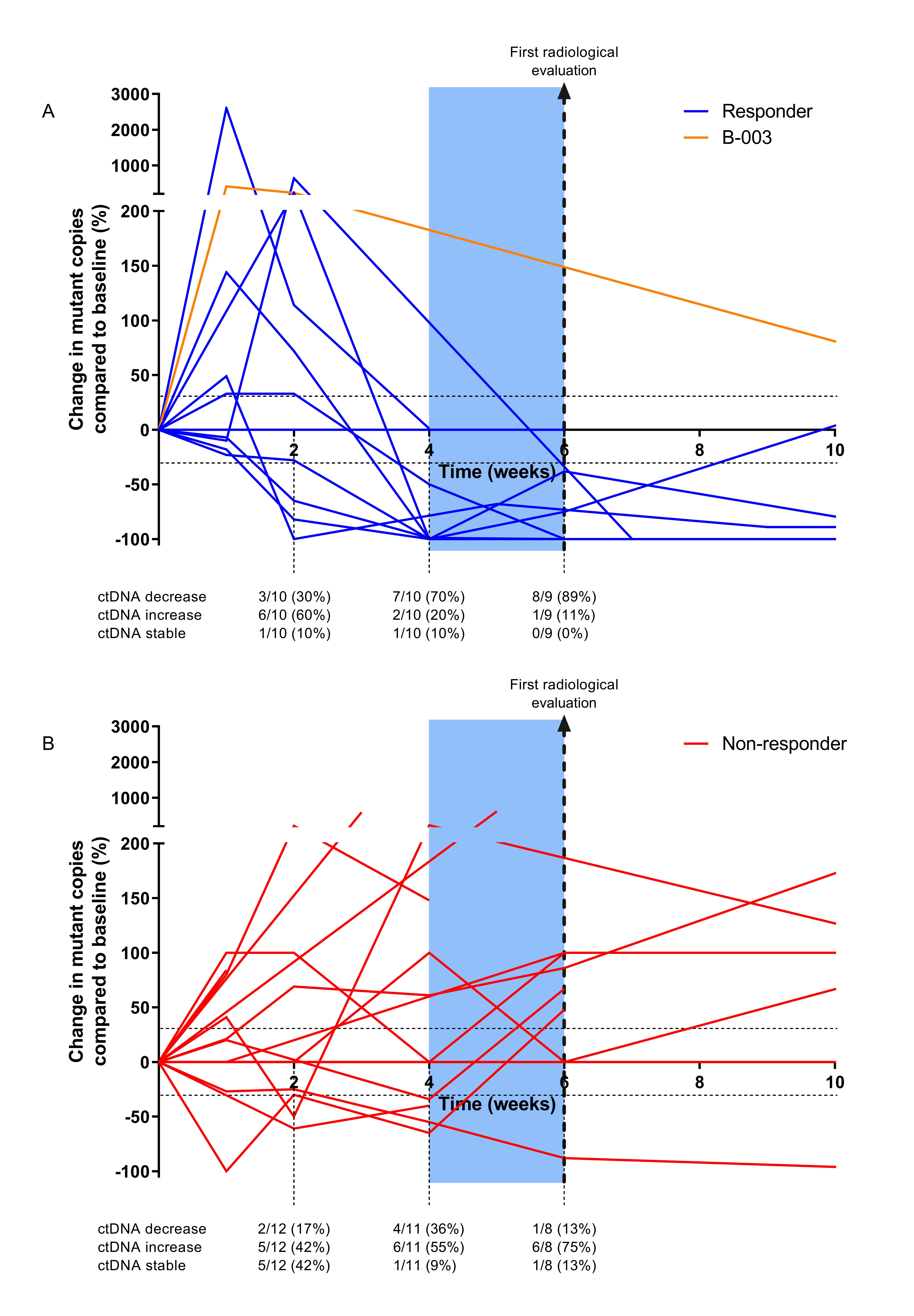
Supplementary Figure 2. Determination of most appropriate timepoints to optimally detect changes in ctDNA levels related to treatment response. Representation of the percentage change in ctDNA levels over time compared to baseline for responders (A, blue) and non-responders (B, red) according to the RECIST v1.1 criteria (see Supplementary Table 1). Dashed lines indicate a 20% increase and 30% decrease in tumor volume compared to baseline. Appropriate timepoints to detect changes in ctDNA prior to the first radiological evaluation are between 4-6 weeks, as most responders have decreased ctDNA levels (A) and most non-responders have increase ctDNA levels (B). Patient B-003 (A, orange) defined as a responder is discordant but lacks plasma samples between 2-12 weeks after treatment for proper interpretation (see Supplementary Figure 3). Data of four patients were not included as mutant ctDNA were not detected (negative cases), one responder and three non-responders.


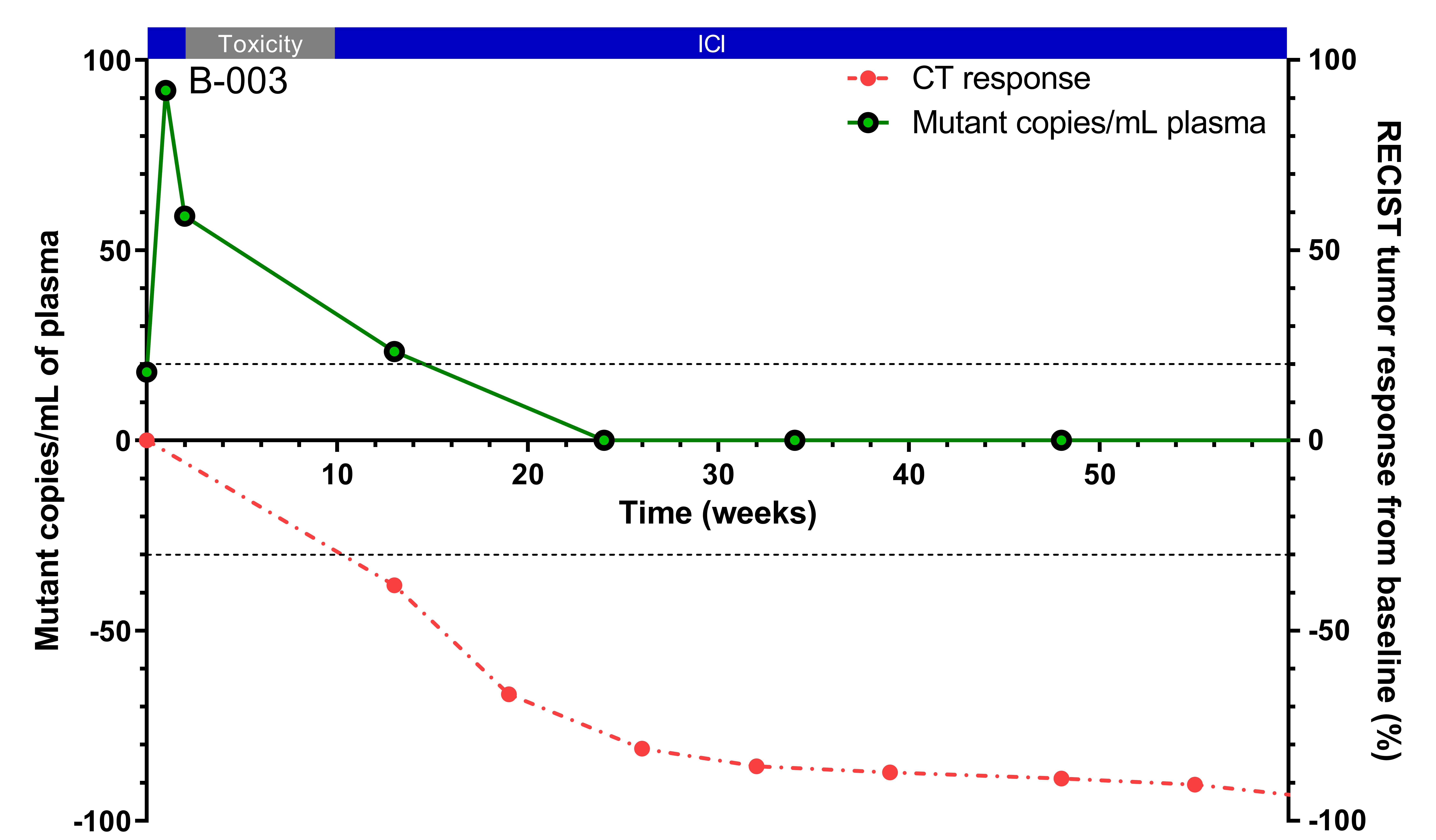
 Supplementary Figure 3. Evaluation of case B-003. Change in mutant copies compared to baseline (t_0_) is presented over time. Dashed lines indicate a 20% increase and 30% decrease in tumor volume compared to baseline. The bar on top indicates the patient received immune checkpoint inhibitors (ICI, blue) the entire follow-up time, except from 2-10 weeks after initiation when treatment was stopped due to toxicity (gray). In this patient, an initial spike in ctDNA was observed in the first two weeks of treatment. No blood sample was collected between 2-12 weeks after start of treatment. At 24 weeks, the patient has complete clearance of ctDNA and showed a consistent response to treatment.


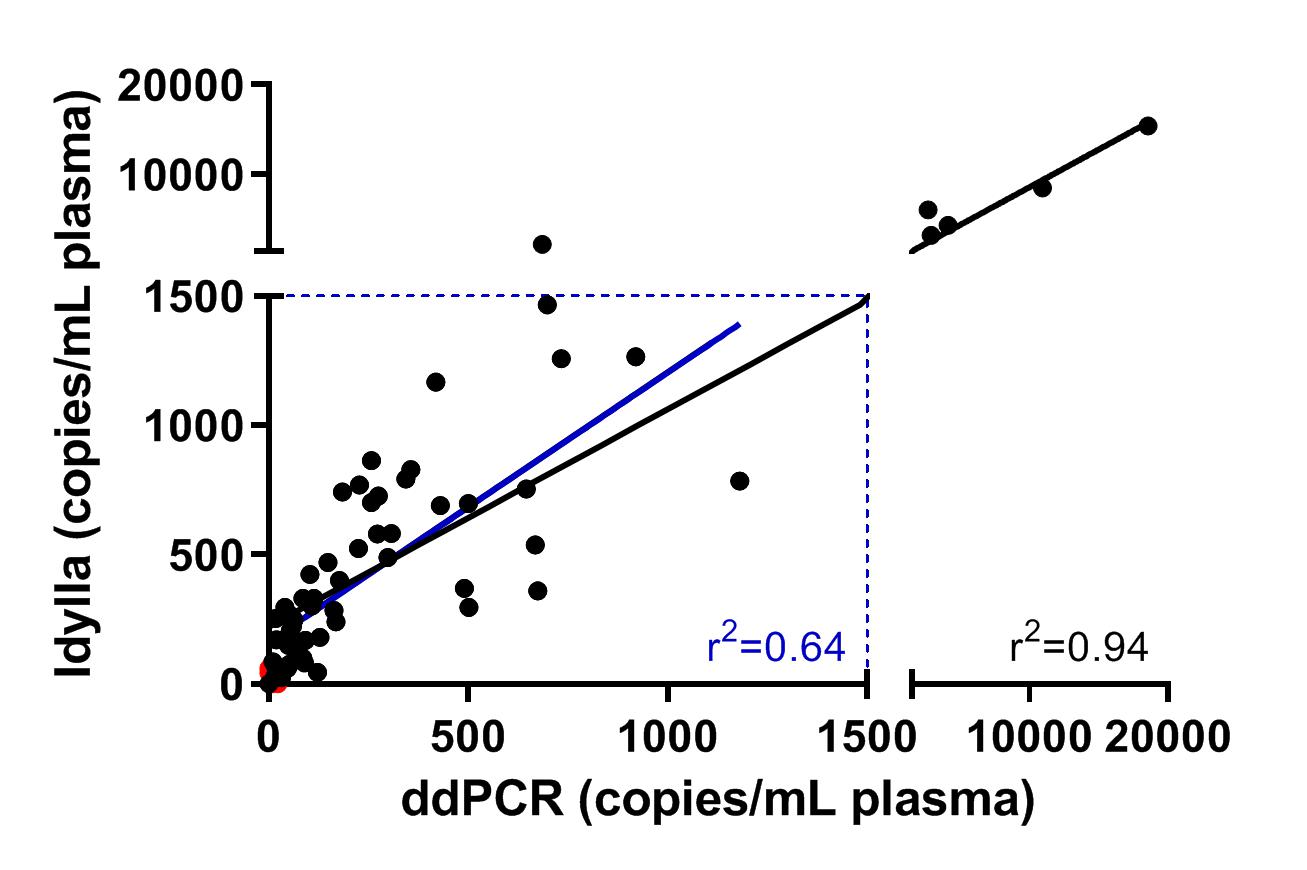
Supplementary Figure 4. Correlation of the mutant copies per mL of plasma as determined with ddPCR and Idylla™ ctKRAS Mutation Assay. Red-colored results were not included in the correlation due to failure of either mutation detection assay. Correlation was determined for all samples (black line, r^2^=0.94) and excluding outliers (blue line, r^2^=0.64).


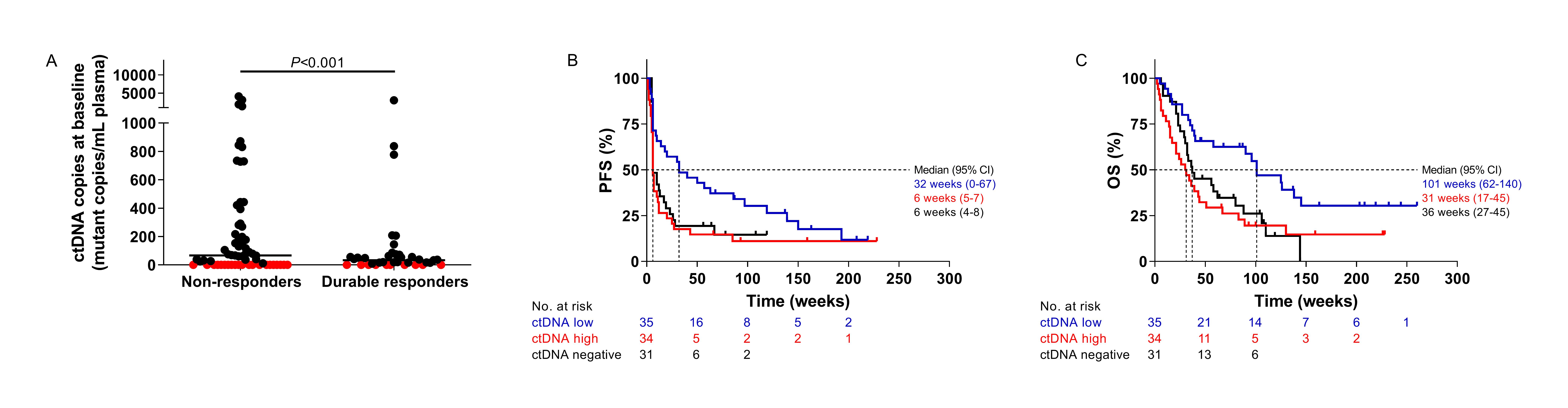
Supplementary Figure 5. Mutant ctDNA levels at baseline. (A) Number of mutant copies per mL of plasma measured prior to start of treatment for patients with no clinical response (<6 months) and durable clinical benefit (≥6 months). Red-colored results were ctDNA negative were excluded from the statistical analysis. These included both non-responders (21/63, 33%) and durable responders (10/37, 27. Kaplan-Meier plots displaying the (B) PFS and (C) OS by separating ctDNA levels at baseline in ctDNA low (below or equal to median levels, *n*=35), ctDNA high (above median, *n*=34) and ctDNA negative (*n*=31). Patients with low and high mutant ctDNA levels at baseline separated significantly with respect to PFS (*P*<0.001) and OS (*P*<0.0001). CI, confidence interval; PFS, progression-free survival; OS, overall survival.


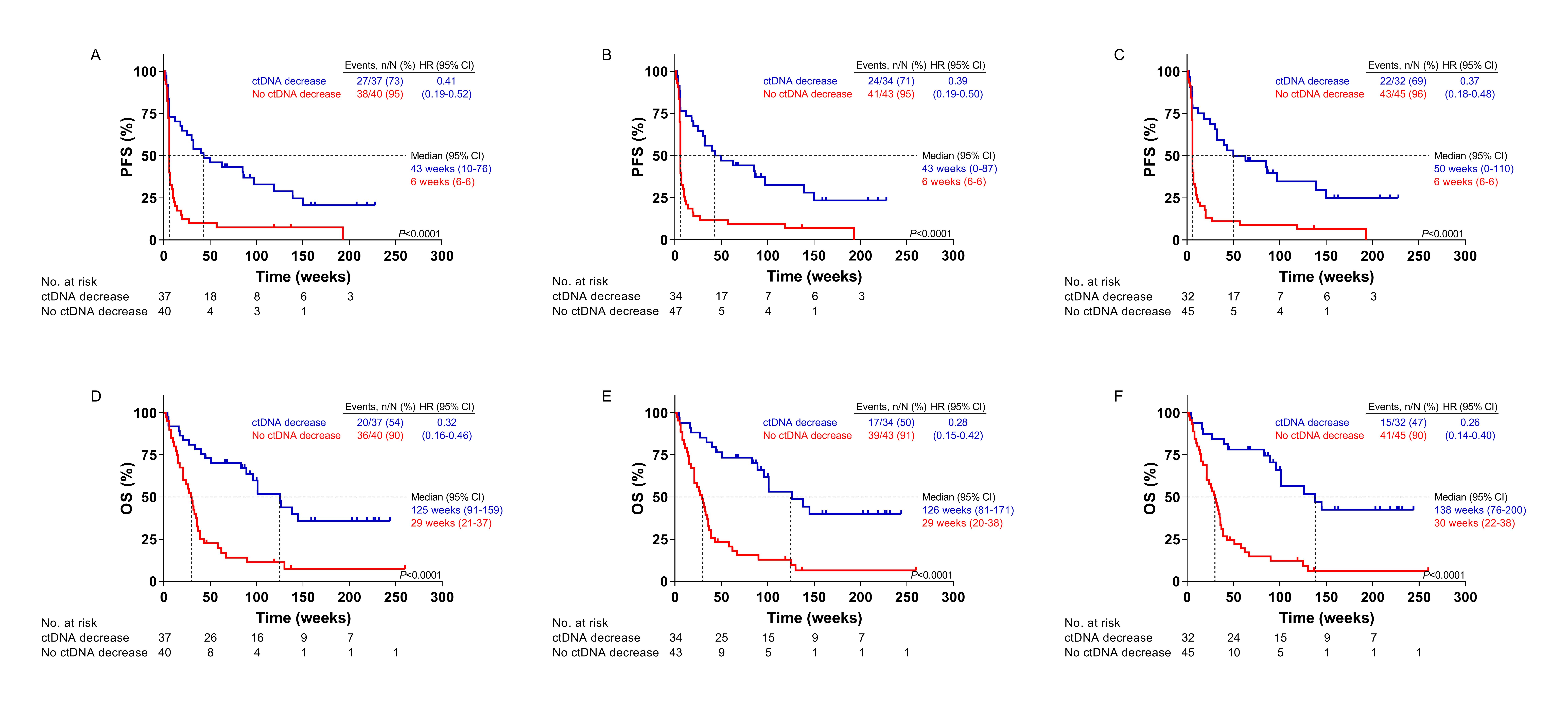
Supplementary Figure 6. PFS and OS at different cut-offs to determine ctDNA decrease. Progression-free survival (A-C) and overall survival (D-F) were analyzed at a 30% (A,D) 40% (B,E) and 50% (C,F) cut-off to determine ctDNA decrease. Increasing the cut-off resulted in slightly lower hazard ratios (HRs), however reduces the number of patients demonstrating a ctDNA decrease. The empirically determined technical cut-off of 30% results in highly significant hazard ratios (P<0.0001) and identifies the most patients with a durable response to ICI treatment.


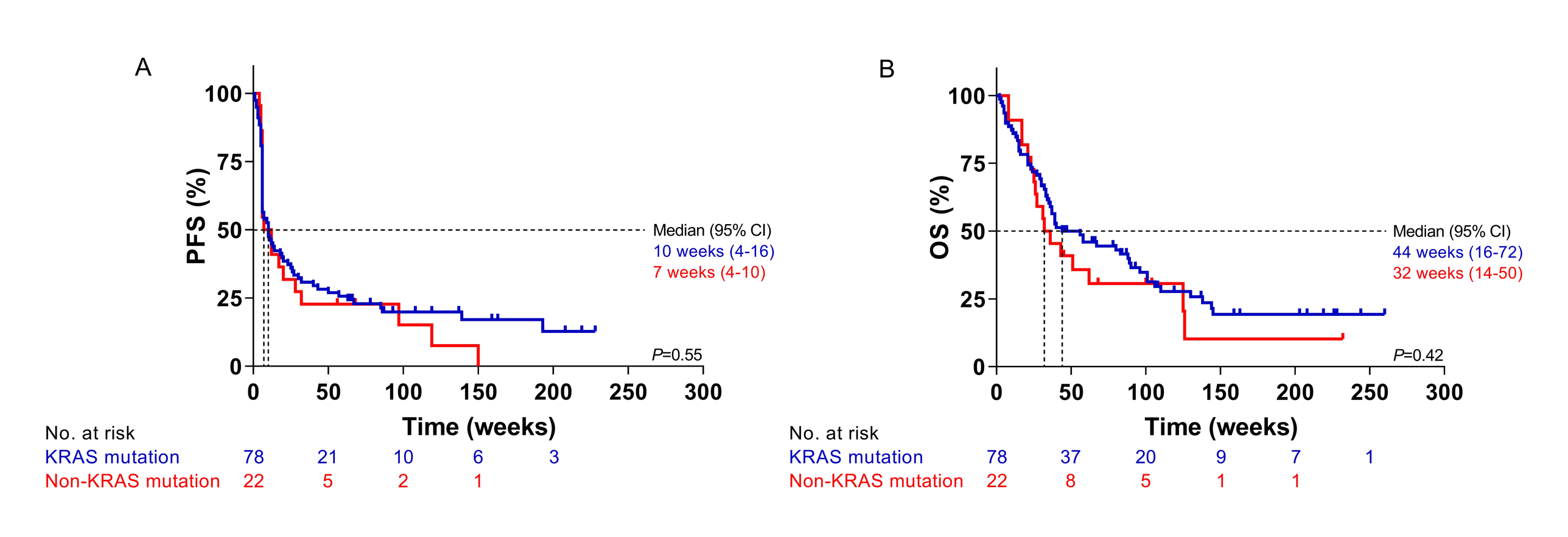
Supplementary Figure 7. PFS and OS is irrespective of *KRAS* mutations. Kaplan-Meier plot displaying the (A) PFS and (B) OS of patients harboring mutations in *KRAS* (blue), or in any other gene detected with ddPCR (red) ctDNA levels. CI, confidence interval; PFS, progression-free survival; OS, overall survival.

#
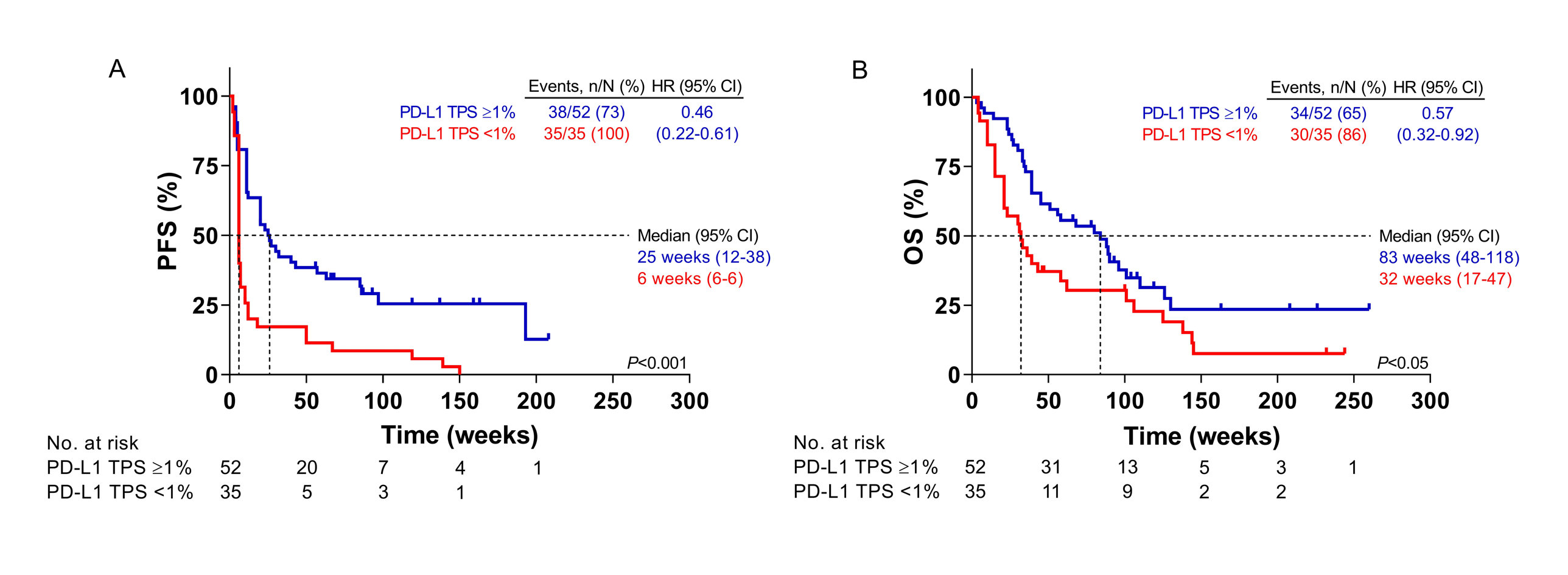
Supplementary Figure 8. Clinical response related to PD-L1 expression. Kaplan-Meier plots displaying the (A) PFS and (B) OS of patients with a tumor PD-L1 expression of <1% (red) or ≥1% (blue). CI, confidence interval; PFS, progression-free survival; OS, overall survival.

#
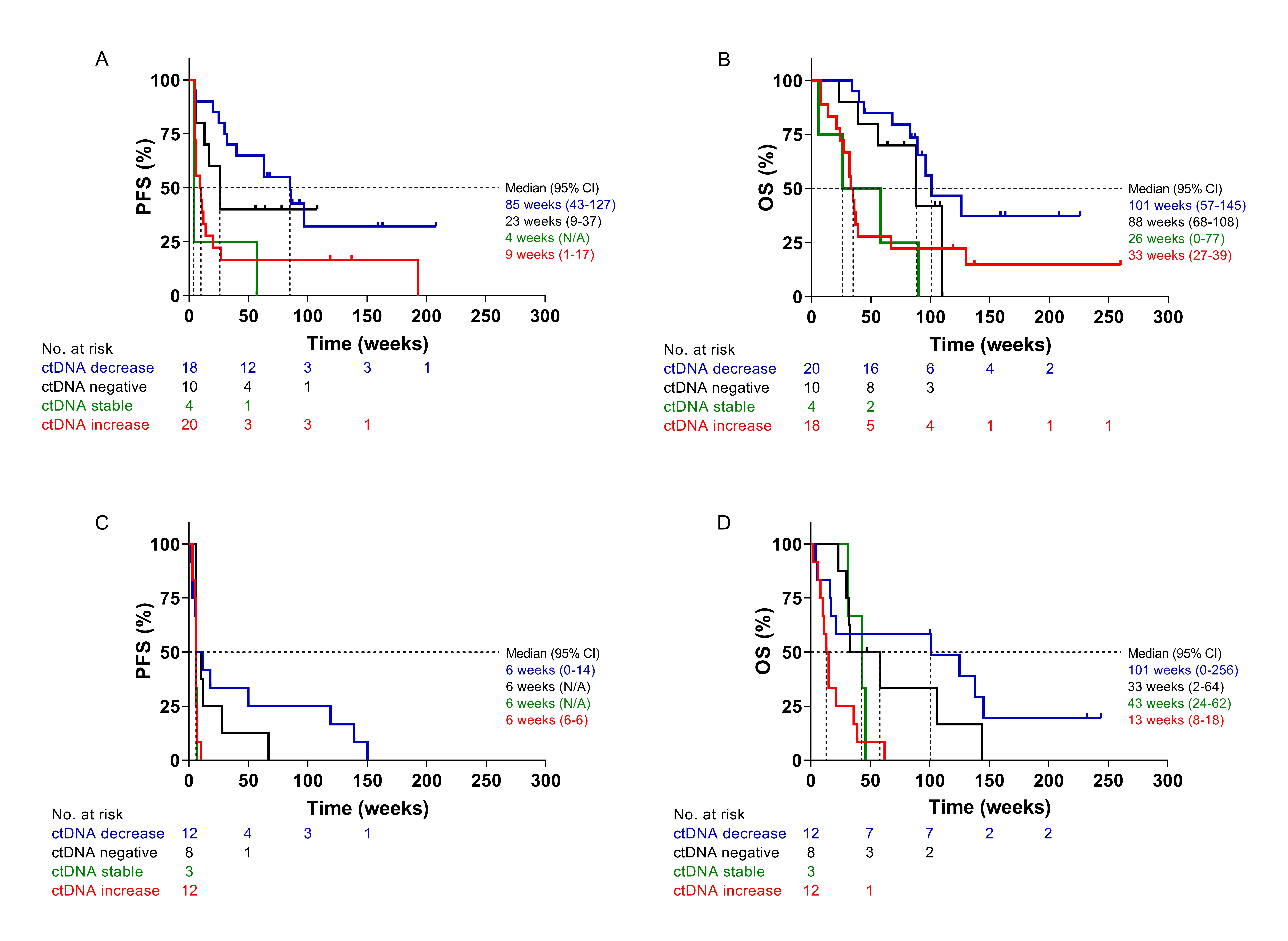
Supplementary Figure 9. Elaborate analysis of radiological response related to PD-L1 expression. Extended representation of clinical response related to change in ctDNA levels in patients with a tumor PD-L1 TPS ≥1% (A-B) and TPS<1% (C-D). Kaplan-Meier plot displaying the (A, C) PFS and (B, D) OS of patients with decreasing (blue), negative (black), stable (green), or increasing (red) ctDNA levels. CI, confidence interval; PFS, progression-free survival; OS, overall survival; TPS, Tumor Proportion Score.

**
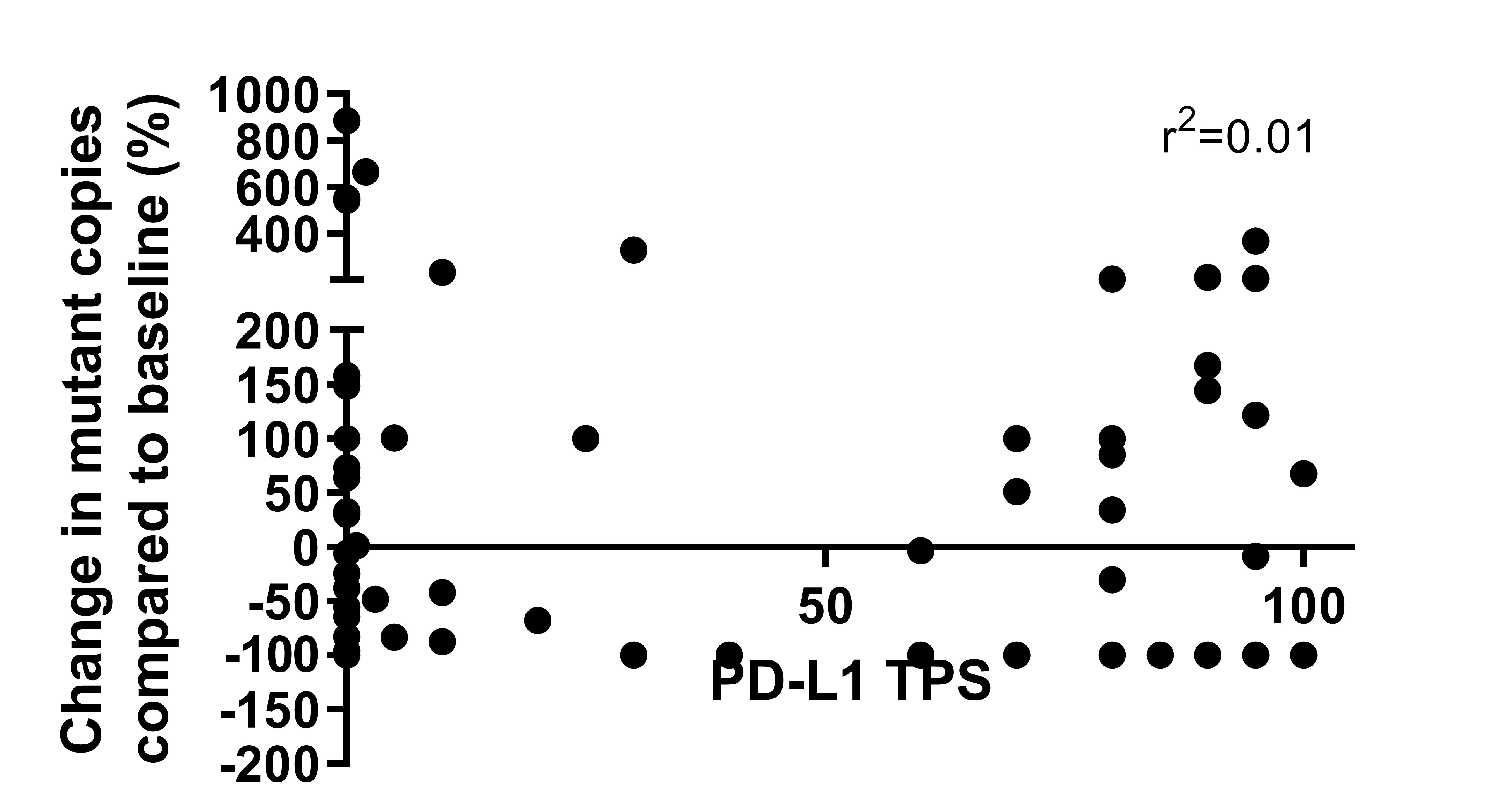
**Supplementary Figure 10. No correlation between change in ctDNA levels and PD-L1 TPS. Pearson’s correlation coefficient, r^2^=0.01 is not considered significant.
